# Supplementary material for: Australian Public Perspectives on Genomic Newborn Screening: Risks, Benefits, and Preferences for Implementation
Source: Int J Neonatal Screen. 2024 Jan 17;10(1):6. doi: 10.3390/ijns10010006 (PMC10801595; doi:10.3390/ijns10010006)
Supplement: Supplementary file 1 [file IJNS-10-00006-s001.zip › IJNS-2744812-supplementary.pdf]

Focus group guide
How should GNBS be implemented?

Welcome

Introductions

Purpose of this Session

Housekeeping

Brief Refresher on Material from the Video

Group Discussion Part 1 – Introductions and warm up questions

Polls

1. Do you think genomics should be used in newborn screening programs?

   Yes No Not sure

2. Should a newborn screening program that includes genomics be run any differently to standard newborn screening programs?

   Yes No Not sure

Group Discussion Part 2

1. Why did you answer yes on no to whether genomics should be used in newborn screening programs?

2. If you answered no to running newborn screening programs that include genomics differently, why?

3. If you answered yes to running newborn screening programs that include genomics differently, what do you think should be done differently?

Now, let's talk about how parents (or prospective parents) should be told about genomic newborn screening.

4. When should genomic newborn screening be initially discussed with parents?
   a. During pregnancy (assess which time points)
   b. At birth
   c. In infancy, e.g. at 3-6 months

5. Who should tell the parents about genomic newborn screening?
   d. GP
   e. Obstetrician

f. Midwife
g. Genetics professional
h. Virtual discussion
i. Other

6. Where should genomic newborn screening be discussed with parents?

j. GP clinic
k. Obstetrician clinic
l. In hospital
m. Online

Let's talk about the way we should ask permission for genomic newborn screening to take place. This is also known as giving consent.

7. Do you think parents need to give consent for genomic newborn screening or should testing happen automatically? Why?

8. Should the consent process be any different from standard newborn screening? Why/why not?

9. When do you think parents should be asked to give consent for genomic newborn screening to take place for their baby?

10. What information do you think is important to know to help parents make decisions about genomic newborn screening for their baby?

11. How should information be provided?

a. Online
b. Written
c. In person discussion

Now let's talk about some of the factors we might want to think about when deciding which genetic conditions to screen for.

12. Does it matter whether the conditions are:

a. Treatable vs non-treatable
    i. treatment that significantly alters natural history (curative);
    ii. treatment that is supportive (e.g. early intervention for intellectual disability);
    iii. early detection can lead to early treatment;
    iv. no approved treatment but clinical trial available;

   v. Does the nature of the treatment matter? E.g. medications vs bone marrow transplant

b. Age of onset of symptoms:
   i. early childhood;
   ii. late childhood-adolescence;
   iii. adulthood
c. Severity:
   i. Death in childhood,
   ii. death in middle age,
   iii. lifelong physical impairment,
   iv. organ failure
   v. lifelong cognitive impairment.
d. Level of certainty that symptoms of the genetic condition will start in childhood
   i. 50%
   ii. 90%
   iii. 100%
e. Level of certainty about what the symptoms of the condition will be (e.g. conditions that invariably cause a particular problem, e.g. bone marrow failure vs conditions that can have a broad range of health issues associated with them but combination and severity in each person varies)

13. What if you could know about many of these conditions before you got pregnant?
    Which types of conditions would you prefer to know about beforehand?
    What would you do with that information?

14. Who should pay for the genomic newborn screening?
    Publicly funded
    Privately funded

Group Discussion Part 3 – Assessing risks and benefits

1. What are your thoughts on whether genomics should be used in newborn screening?
2. What do you see as some of the advantages of using genomics in newborn screening?
3. What do you see as some of the potential risks of using genomics in newborn screening?
4. What do you think is important when thinking about using genomics in newborn screening? (just to list a few examples to prompt)
   - Types of conditions
   - Giving consent
   - Payment
5. Do you think genomic data should be stored after it has been used in screening?
   - For programme evaluation?

    - For future medical use of person being screening?
    - For medical research?
6. Is there anything else we have missed?

Polls

1. Do you think genomics should be used in newborn screening programs?

    Yes No Not sure

2. Should a newborn screening program that includes genomics be run any differently to standard newborn screening programs?

    Yes No Not sure

Wrap up

- Final comments and thoughts from participants
- Summary of discussions
- Next steps
